# Anti-embolism devices therapy to improve the ICU mortality rate of patients with acute myocardial infarction and type II diabetes mellitus

**DOI:** 10.3389/fcvm.2022.948924

**Published:** 2022-07-19

**Authors:** Xiaxuan Huang, Luming Zhang, Mengyuan Xu, Shiqi Yuan, Yan Ye, Tao Huang, Haiyan Yin, Jun Lyu

**Affiliations:** ^1^Department of Neurology, The First Affiliated Hospital of Jinan University, Guangzhou, China; ^2^Department of Clinical Research, The First Affiliated Hospital of Jinan University, Guangzhou, China; ^3^Department of Intensive Care Unit, The First Affiliated Hospital of Jinan University, Guangzhou, China; ^4^Guangdong Provincial Key Laboratory of Traditional Chinese Medicine Informatization, Guangzhou, China

**Keywords:** anti-embolic therapy, acute myocardial infarction, type II diabetes mellitus, mortality, ICU

## Abstract

**Background:**

Anti-Embolism (AE) devices therapy is an additional antithrombotic treatment that is effective in many venous diseases, but the correlations between this medical compression therapy and cardiovascular arterial disease or comorbid diabetes mellitus (DM) are still controversial. In this study we investigated the association between compression therapy and intensive care unit (ICU) mortality in patients with a first acute myocardial infarction (AMI) diagnosis complicated with type II DM.

**Methods:**

This retrospective cohort study analyzed all patients with AMI and type II DM in the Medical Information Mart for Intensive Care-IV database. We extracted the demographics, vital signs, laboratory test results, comorbidities, and scoring system results of patients from the first 24 h after ICU admission. The outcomes of this study were 28-day mortality and ICU mortality. Analyses included Kaplan–Meier survival analysis, Cox proportional-hazards regression, and subgroup analysis.

**Results:**

The study included 985 eligible patients with AMI and type II DM, of who 293 and 692 were enrolled into the no-AE device therapy and AE device therapy groups, respectively. In the multivariate analysis, compared with no-AE device therapy, AE device therapy was a significant predictor of ICU mortality (HR = 0.48, 95% CI = 0.24–0.96, *P* = 0.039) and 28-day mortality (HR = 0.50, 95% CI = 0.27–0.90, *P* = 0.021). In addition to age, gender and coronary artery bypass grafting surgery, there were no significant interactions of AE device therapy and other related risk factors with ICU mortality and 28-day mortality in the subgroup analysis.

**Conclusions:**

Simple-AE-device therapy was associated with reduced risks of ICU mortality and 28-day mortality, as well as an improvement in the benefit on in-hospital survival in patients with AMI complicated with type II DM.

## Introduction

The prevalence of type II diabetes mellitus (DM) is increasing greatly worldwide. The number of patients with diabetes has been predicted to increase to 300 million by 2025 (1). DM is currently the most-serious factor contributing to heart failure and reinfarction prognoses caused by acute myocardial infarction (AMI) in cardiovascular diseases. It has been classified as a marker of a poor prognosis after AMI (2, 3). AMI caused by type II DM is the complication with the highest mortality and disability rate among diabetes-induced diseases, which has received widespread attention from both governments and the general public. Back in 2010, the NATIONAL Institute for Health and Clinical Excellence (NICE) guidelines recommended the combined use of mechanical prophylaxis (AES, foot impulse devices, intermittent pneumatic compression devices), whether a mechanical device used to prevent venous thromboembolism, as a simple non-invasive medical device, can also prevent ICU patients with acute myocardial infarction complicated with diabetes who lack certain activity, is worth exploring and studying its possibility (4, 5).

During the course of the continuous disease progression, patients with diabetes and AMI are prone to large or small microvascular neuropathies and cardiomyopathies due to coronary heart disease (6). Especially under the conditions of high blood sugar, along with the vascular endothelial cell injury, lipid deposition in macrophages and cause atherosclerosis, vascular smooth-muscle proliferation, and blood high condensation conditions to thrombosis, easy to increase vascular stenosis or blocked, so as to make the myocardial ischemia in partial necrosis (7, 8), eventually leading to heart failure or life-threatening cardiac shock. Thus, the early prevention of lower limb vein edema and periodic peripheral edema therefore appears to be particularly important in controlling the incidence of complications (9, 10). In anti-embolism (AE) treatment, which includes the removal of anticoagulation, thrombi therapy, and simple-AE-device auxiliary therapy (11), simple-AE-device therapy (including elastic stockings, ACE wraps, and compression sleeves) (4, 12, 13) is generally made most patients with diabetes and AMI more compliant than other early anti-embolism interventions, which is due to its simple, non-invasive, and convenient characteristics, and the ability to wear the device and use it in daily life. Most (90%) of patients with diabetes and AMI can improve their survival by enhancing the compression of the package to dissolve the fibrin discharged from the vein to relieve swelling (14, 15).

However, AE device therapy has long been controversial due to concern about endangering arterial circulation under high pressures (7, 16, 17). In order to solve this problem, this study compared the risk of type II DM with other established risk factors for death. The results suggested that simple-AE-device therapy has a specific prognostic impact on patients with type II DM and AMI. This retrospective cohort study examined the associations of AE device therapy alone with ICU mortality and 28-day mortality in type II DM with AMI.

## Methods

### Data source

A large, single-center, public database called the Medical Information Mark for Intensive Care (MIMIC-IV) (18–20) was used in this study. It was approved by the Massachusetts Institute of Technology (Cambridge, MA) and Beth Israel Deaconess Medical Center (Boston, MA). Because the present study performed an analysis of third-party anonymized publicly available data with pre-existing institutional review board (IRB) approval, approval from the IRB of our institution was not needed. In the database, the true identity information of the patients are hidden. Obtaining informed consent from patients was therefore not needed. After completing the online course of the National Institutes of Health and passing the examination for the protection of human study participants, all of the authors obtained a certificate to access the database (record ID: 45351934).

### Population selection criteria

**Figure 1:**
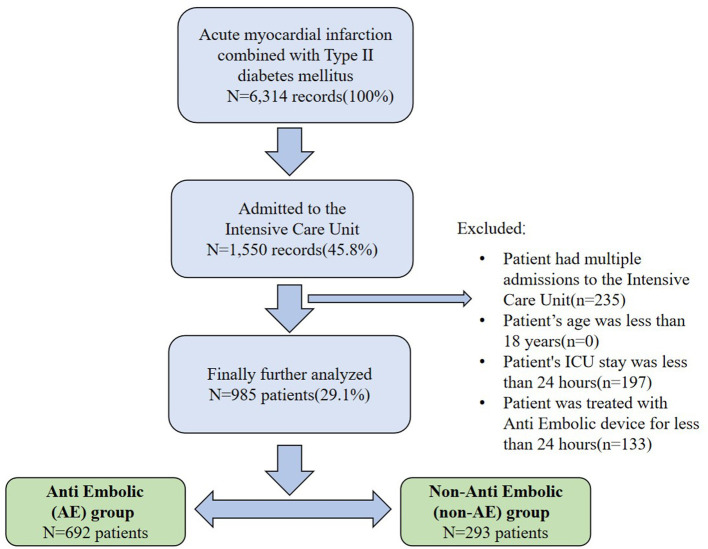
Flowchart of study population selection.

According to ICD-9 and ICD-10 codes, a total of 6,314 AMI records were included in MIMIC-IV database, including 3,383 records of patients with type II diabetes. Patients were excluded based on the following criteria: (1) multiple ICU admissions, (2) younger than 18 years, (3) ICU stay shorter than 24 h, or (4) treated using an AE device for <24 h. The follow-up duration was 28 days after the time of admission, and the survival status was observed at discharge. The final cohort included 985 patients, 293 and 692 of who were enrolled into the no-AE device therapy and AE device therapy groups, respectively. According to the above inclusion criteria, we extracted relevant information using Structured Query Language (SQL) in the Navicat Premium (version 15.0) program by identifying the subject_ids of the study population. The flow chart of included patients is illustrated in Figure 1.

### Data extraction

Data were extracted using SQL with PostgreSQL tools (version 15.0). Extracted data included demographics, vital signs, comorbidities, llaboratory tests within the first 24 h of ICU admission. The initially selected laboratory measurements included age, sex, weight, Acute Physiology Score III (APSIII), ethnicity, first care unit, ventilator and vasopressor use, continuous renal replacement therapy (CRRT) use, percutaneous coronary intervention (PCI), coronary artery bypass grafting (CABG), antiplatelets, anticoagulation, hypertension, congestive heart failure, peripheral vascular disease, cerebrovascular disease, chronic pulmonary disease, renal disease, liver disease, malignant cancer, mean blood pressure (MBP), heart rate, respiratory rate, mean oxygen saturation (SpO_2_), temperature, maximum troponin T, creatine kinase-myocardial band (CKMB), white blood cells (WBCs), hemoglobin, platelets, potassium, creatinine, blood urea nitrogen, maximum glucose, international normalized ratio (INR), alanine transaminase (ALT), urine output, anion gap (AG), and lactate. The endpoints of our study were 28-day mortality and ICU mortality.

### Statistical analysis

The covariates of the no-AE device therapy and AE device therapy groups were compared using the chi-square or Fisher's exact tests as appropriate. Continuous variables are represented by mean and standard deviations or medians and interquartile ranges (IQR) (21, 22).

Subgroup analyses were performed to access the associations of AE device therapy with 28-day mortality and ICU mortality, and included age, sex, PCI, CABG, antiplatelets, and anticoagulation therapy. The data were analyzed using R software (http://www.R-project.org). Cox proportional-hazards regression models with increasing covariates were established to analyze the effects of multiple factors on survival time and clinical status. Kaplan–Meier survival analysis was used to examine differences in ICU mortality among groups. Log-rank tests were used to further compare differences among groups. A probability value of *P* < 0.05 was considered statistically significant, and all probability values were two-sided.

## Results

### Baseline results

This study enrolled 985 eligible patients, of whom 875 were survivors and 110 were non-survivors. The characteristics of the patients in the no-AE device therapy and AE device therapy groups are summarized in Table 1. Differences were found between the groups in the first care unit, ventilator and vasopressor use, PCI, CABG, antiplatelets, anticoagulation, cerebrovascular disease, MBP, mean heart rate, mean SpO_2_, maximum troponin T, CKMB, WBCs, hemoglobin, glucose, ALT, urine output, and lactate (*P* < 0.05).

**Table 1:** Baseline characteristics of the study population.

	**Non-AE-therapy**	**AE-therapy**	***p*** **-value**
***N***	**293**	**692**	
**Age, years**	**71.00 (63.00, 80.00)**	**71.50 (63.00, 79.00)**	**0.863**
**Gender, *n* (%)**			**0.401**
**Male**	**173 (59.0)**	**430 (62.1)**	
**Female**	**120 (41.0)**	**262 (37.9)**	
**Weight, kg**	**86.05 (72.90, 99.00)**	**84.10 (71.12, 97.68)**	**0.218**
**APSIII**	**44.00 (35.00, 61.00)**	**49.00 (36.00, 68.00)**	**0.003**
**Ethnicity, *n* (%)**			**0.988**
**White**	**174 (59.4)**	**413 (59.7)**	
**Others**	**119 (40.6)**	**279 (40.3)**	
**First careunit, *n* (%)**			** <0.001**
**CCU**	**266 (90.8)**	**449 (64.9)**	
**Others**	**27 (9.2)**	**243 (35.1)**	
**Ventilator, *n* (%)**			** <0.001**
**No**	**204 (69.6)**	**230 (33.2)**	
**Yes**	**89 (30.4)**	**462 (66.8)**	
**Vasopressor, *n* (%)**			**0.001**
**No**	**214 (73.0)**	**426 (61.6)**	
**Yes**	**79 (27.0)**	**266 (38.4)**	
**CRRT, *n* (%)**			**0.719**
**No**	**288 (98.3)**	**676 (97.7)**	
**Yes**	**5 (1.7)**	**16 (2.3)**	
**PCI, *n* (%)**			** <0.001**
**No**	**128 (43.7)**	**601 (86.8)**	
**Yes**	**165 (56.3)**	**91 (13.2)**	
**CABG, *n* (%)**			** <0.001**
**No**	**290 (99.0)**	**438 (63.3)**	
**Yes**	**3 (1.0)**	**254 (36.7)**	
**Antiplatelet, *n* (%)**			**0.028**
**No**	**7 (2.4)**	**41 (5.9)**	
**Yes**	**286 (97.6)**	**651 (94.1)**	
**Anticoagulation, *n* (%)**			**0.008**
**No**	**20 (6.8)**	**89 (12.9)**	
**Yes**	**273 (93.2)**	**603 (87.1)**	
**Hypertension, *n* (%)**			**0.589**
**No**	**173 (59.0)**	**423 (61.1)**	
**Yes**	**120 (41.0)**	**269 (38.9)**	
**Congestive heart failure, *n* (%)**			**0.879**
**No**	**114 (38.9)**	**264 (38.2)**	
**Yes**	**179 (61.1)**	**428 (61.8)**	
**Peripheral vascular disease, *n* (%)**			**0.426**
**No**	**241 (82.3)**	**585 (84.5)**	
**Yes**	**52 (17.7)**	**107 (15.5)**	
**Cerebrovascular disease, *n* (%)**			**0.002**
**No**	**259 (88.4)**	**552 (79.8)**	
**Yes**	**34 (11.6)**	**140 (20.2)**	
**Chronic pulmonary disease, *n* (%)**			**1.000**
**No**	**220 (75.1)**	**519 (75.0)**	
**Yes**	**73 (24.9)**	**173 (25.0)**	
**Renal disease, *n* (%)**			**0.563**
**No**	**170 (58.0)**	**386 (55.8)**	
**Yes**	**123 (42.0)**	**306 (44.2)**	
**Liver disease, *n* (%)**			**0.962**
**No**	**269 (91.8)**	**633 (91.5)**	
**Yes**	**24 (8.2)**	**59 (8.5)**	
**Malignant cancer, *n* (%)**			**0.136**
**No**	**280 (95.6)**	**642 (92.8)**	
**Yes**	**13 (4.4)**	**50 (7.2)**	
**Mbp mean, mmHg**	**76.88 (69.31, 83.67)**	**74.64 (69.71, 79.77)**	**0.016**
**Heart rate mean, beats/min**	**79.51 (70.50, 88.00)**	**83.19 (73.88, 91.72)**	** <0.001**
**Respiratory rate mean, beats/min**	**19.04 (17.24, 21.21)**	**19.03 (16.95, 21.09)**	**0.516**
**SpO_2_ mean, %**	**96.63 (95.36, 97.96)**	**97.33 (96.09, 98.50)**	** <0.001**
**Temperature mean, °C**	**36.79 (36.61, 36.95)**	**36.80 (36.60, 37.02)**	**0.529**
**Troponin T Max, ng/mL**	**2.17 (0.80, 5.80)**	**0.86 (0.27, 2.74)**	** <0.001**
**CKMB, ng/mL**	**21.00 (6.00, 75.75)**	**9.00 (4.00, 26.00)**	** <0.001**
**WBC, K/uL**	**10.70 (8.00, 14.20)**	**9.70 (7.60, 13.20)**	**0.011**
**Hemoglobin, g/dl**	**11.20 (9.80, 12.95)**	**10.80 (9.10, 12.40)**	**0.003**
**Platelet, K/uL**	**208.50 (163.00, 263.75)**	**199.00 (150.00, 254.50)**	**0.052**
**Potassium, mEq/L**	**4.30 (3.90, 4.70)**	**4.30 (3.90, 4.70)**	**0.612**
**Creatinine, mg/dL**	**1.30 (0.90, 2.00)**	**1.30 (0.90, 2.02)**	**0.792**
**Urea Nitrogen, mg/dL**	**26.50 (17.00, 40.00)**	**26.00 (17.00, 43.00)**	**0.976**
**Glucose max, mg/dl**	**254.50 (194.75, 329.00)**	**221.00 (186.75, 280.00)**	** <0.001**
**INR**	**1.20 (1.10, 1.40)**	**1.20 (1.10, 1.40)**	**0.580**
**ALT, IU/L**	**31.00 (18.00, 73.25)**	**26.00 (16.00, 48.00)**	**0.002**
**Urine output, ml/s**	**1720.00 (1075.00, 2465.00)**	**1450.00 (911.50, 2191.25)**	**0.003**
**Anion Gap, mEq/L**	**16.00 (14.00, 20.00)**	**16.00 (14.00, 19.00)**	**0.565**
**Lactate, mmol/L**	**1.90 (1.30, 2.70)**	**1.60 (1.20, 2.40)**	**0.002**
**Day 28-mortality, *n* (%)**			
**0**	**240 (81.9)**	**598 (86.4)**	**0.086**
**1**	**53 (18.1)**	**94 (13.6)**	
**ICU-mortality, *n* (%)**			
**0**	**252 (86.0)**	**623 (90.0)**	**0.085**
**1**	**41 (14.0)**	**69 (10.0)**	


### Kaplan–meier survival curve analysis

**Figure 2:**
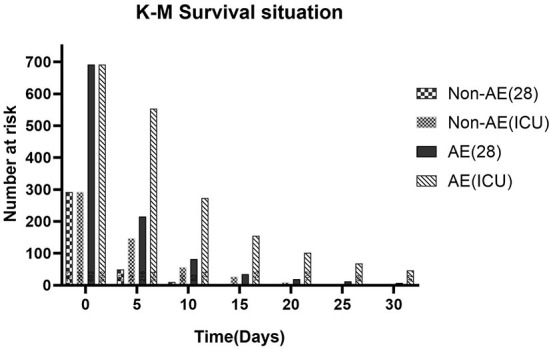
K–M survival situation about AE-device therapy and outcomes at different times.

The Kaplan–Meier curves of all examined categorical variables are illustrated in Figure 2. Those in the AE device therapy group had a higher probability of survival than did those in the no-AE device therapy group. We analyzed the different survival conditions between the two groups according to the time nodes obtained using the Kaplan–Meier survival curve. Survival at 28 days and in the ICU was significantly more likely in the AE device therapy group than in the no-AE device therapy group when the follow-up time was changed.

### Cox proportional-hazards models

A Cox proportional-hazards regression model was constructed to further investigate the effects of multiple variables on survival time and outcome, and to estimate the hazard ratios (HR) for 28-day mortality and ICU mortality. As listed in Table 2, compared with no-AE device therapy, AE device therapy was a significant predictor of ICU mortality (HR = 0.48, 95% CI = 0.24–0.96, *P* = 0.039) and 28-day mortality (HR = 0.50, 95% CI = 0.27–0.90, *P* = 0.021) after adjusting for covariates.

**Table 2:** Analysis of the associations between AE-device therapy and outcomes.

	**Non-AE-device therapy**	**AE-device therapy**	***p*-value**
	**HR (95%CI)**	**HR (95%CI)**	
**ICU-mortality**			
Unadjusted	Reference	0.32 (0.22, 0.48)	<0.001
Adjusted	Reference	0.48 (0.24, 0.96)	0.039
**Day28 mortality**			
Unadjusted	Reference	0.44 (0.31, 0.62)	<0.001
Adjusted	Reference	0.50 (0.27, 0.90)	0.021

*Analysis of the associations between AE-device therapy and outcomes*.

*HR, hazard ratio; CI, confidence interval*.

*Models were derived from Cox proportional hazards regression models*.

*Model I was not adjusted for covariates*.

*Model II covariates were adjusted for Age, Weight, Ethnicity, Gender, First_careunit, APSIII, Anion_Gap, Heart_rate_mean, CKMB, WBC, Respiratory_rate_mean, Mbp_mean, SpO_2__mean, Temperature_mean, Troponin_T_Max, Hemoglobin, Glucose_max, INR, Platelet, Potassium, Creatinine, Urea_Nitrogen, ALT, Urine_output, Lactate, Anti_Embolic, Antiplatelet, Anticoagulation, Congestive_heart_failue, Renal_disease, Malignant_cancer, Liver_disease, PCI, CABG, Ventilator, Vasopressor, CRRT, Peripheral_vascular_disease, Cerebrovascular_disease, Chronic_pulmonary_disease, Hypertensionid*.

### Subgroup analyses

A subgroup analysis was applied to the main influencing variables in this study to determine the associations of AE device therapy with 28-day mortality and ICU mortality (Table 3). There were no significant interactions in most strata in the subgroup analyses (*P* = 0.146). Nevertheless, patients who were ≥65 years old, female, and received PCI, CABG, or anticoagulation (heparin and warfarin) therapy had significantly higher risks of 28-day mortality and ICU mortality.

**Table 3:** Subgroup analysis of the associations between AE-therapy and outcomes.

	**ICU-mortality**		**Day 28-mortality**	
	**HR (95%CI)**	***p*** **-value**	**HR (95%CI)**	***p*** **-value**
**Age, years**
** ≤ 65 (*n* = 300)**	**NA**		**NA**	
**>65 (*n* = 685)**	**0.32 (0.13, 0.77)**	**0.010**	**0.35 (0.17, 0.71)**	**0.003**
**Gender, *n* (%)**
**Male (*n* = 603)**	**0.52 (0.21, 1.31)**	**0.164**	**0.57 (0.27, 1.21)**	**0.146**
**Female (*n* = 382)**	**0.02 (0.01, 0.04)**	** <0.001**	**0.06 (0.03, 0.12)**	** <0.001**
**PCI, *n* (%)**
**No (*n* = 729)**	**0.42 (0.15, 1.15)**	**0.092**	**0.43 (0.20, 0.90)**	**0.025**
**Yes (*n* = 256)**	**NA**		**NA**	
**CABG, *n* (%)**
**No (*n* = 728)**	**0.47 (0.23, 0.95)**	**0.036**	**0.50 (0.28, 0.91)**	**0.023**
**Yes (*n* = 257)**	**NA**		**NA**	
**Antiplatelet, *n* (%)**
**No (*n* = 48)**	**NA**		**NA**	
**Yes (*n* = 937)**	**0.67 (0.32, 1.42)**	**0.302**	**0.58 (0.31, 1.09)**	**0.093**
**Anticoagulation, *n* (%)**
**No (*n* = 109)**	**NA**		**NA**	
**Yes (*n* = 876)**	**0.49 (0.24, 1.02)**	**0.058**	**0.47 (0.25, 0.89)**	**0.020**

*Subgroup analysis of the associations between AE-therapy and outcomes*.

*Hazard ratio (95% CI): from Cox proportional hazards regression models. The covariate adjustment was consistent with Model II in Table 2*.

## Discussion

As a strong risk factor for cardiovascular disease, type II DM (1, 23) is a common clinical endocrine and metabolic disease that is primarily characterized by hyperglycemia. Long-term hyperglycemia caused by impaired insulin secretion leads to the chronic dysfunction of various tissues, and sustained ischemia and hypoxia leads to myocardial necrosis of varying degrees. Due to the greatly damaged vascular endothelial cells of patients with high blood glucose, lipid deposition in the vascular wall leads to arteriosclerosis. Increased platelet adhesion often results in blood in the hypercoagulable state becoming thrombotic in arterioles, thus blocking blood vessels and aggravating luminal stenosis (24–26). Therefore, in patients with AMI and DM—who are prone to coronary artery branch stenosis or intramyocardial coronary artery stenosis—a collateral circulation disorder can cause extensive infarction, making early preventive AE device therapy for this population particularly important. Therefore, simple-AE-device treatment has received considerable attention in recent cardiovascular research (27).

Considering that AE-device therapy is closely associated with AMI complicated with DM, we selected 985 CCU patients from a large critical-care database (MIMIC-IV) and adjusted for numerous potential confounders, including APSIII, CRRT, CABG, and PCI. Survival appeared to be more likely in the AE-therapy group than in the no-AE device therapy group, with significant differences in ICU mortality and 28-day mortality. Below we summarize the findings and contributions made.

In the past 30 years, some large randomized clinical trials have shown that the main methods to prevent and treat AMI caused by venous thromboembolism include Catheter-directed thrombolysis and endovascular treatment (28, 29), which can clear the disease to a certain extent, greatly reduce the burden of thrombosis, protect vascular and valve functions, and thus reduce the incidence of recurrence of myocardial infarction. However, in actual clinical applications (30), because there are very strict indications and contraindications for thrombolysis or endovascular therapy in patients with deep vein thrombosis (DVT), especially in patients with diabetes, while AE device therapy is non-invasive and convenient, a certain degree of compression can ameliorate limb pain and swelling in DVT ideally, thus greatly improving the compliance of patients, which can allow treatment alongside daily activities as well. This finding was similar to the results of the present study, suggesting that applying therapy with a simple embolization device to patients with AMI and DM can influence mortality outcomes. A subgroup analysis indicated that female patients older than 65 years often have adverse cardiovascular outcomes compared with the remaining AMI population. Especially for diabetic patients with a history of CABG and PCI who have taken anticoagulant drugs for a long time, using AE device therapy can protect the early prognosis of patients, which has definite clinical significance (31).

Previous studies (31, 32) have shown that venous valve function can be improved by applying pressure to leg tissues and blood vessels to support blood pumping to the calf muscles, speeding blood flow back from the legs to the heart, and reducing the risk of thrombosis and embolism. Based on the present study, considering the changes of platelet and coagulation function in patients with AMI combined with DM and the increased risk of thrombosis, AE device therapy is of great significance for these patients, and can greatly reduce the risk of recurrent myocardial infarction or adverse cardiovascular outcomes at an early stage. Simple compression devices (4, 12, 33) including elastic stockings, ACE wraps, and compression sleeves are well-suited for patients with diabetes and AMI, and mild compression can provide them with a certain degree of comfort. In contrast, AE devices have long been clinically controversial because of the risk to the arterial circulation caused by long-term high pressure compression. The long-term application of high degrees of compression may indeed lead to ischemic skin injury, and may even cause accidental injury (16, 34). We therefore compared different use durations and clinical stasus of patients using simple AE devices. As the Kaplan–Meier Survival Curve analysis showed that these devices were most commonly used for 0–20 days, with the longest use time not exceeding 3 months. The use of simple AE compression devices must be accompanied by appropriate management and monitoring, and interventions should be performed in the early stages of discomfort or ischemic injury so as to remove patient concerns about the potential risks of AE device therapy and improve the compliance of patients with AMI complicated with DM using this treatment method. It has to be mentioned that this study is the first to focus on ICU patients with AMI with type II diabetes from the perspective of adjuvant therapy, which can not only improve the compliance of ICU patients, but also provide a new reference for how to effectively support the prognostic treatment of patients with diabetes complicated with AMI.

## Limitations

This was the first study of the correlation between AE device therapy and AMI complicated with DM. However, this study did have some limitations. First, the study had a single-center retrospective design, and so there was selection bias for the population and covariate factors, which would be overcome by a prospective multicenter design. Second, we only extracted certain laboratory indicators and scores from patients with AMI complicated with DM who were admitted to ICUs, and did not analyze the dynamic changes of indicators and scores, which could directly reflect the prognosis of patients. Third, the MIMIC-IV database lacks the different use times of specific simple-AE-device therapies, so it was not possible to compare the efficacy of AE device therapy among different populations, which means that the study was not detailed or comprehensive. Fourth, as a retrospective study, the number of patients included was not large, which means that there are many uncertainties when attempting to generalize its conclusions to other populations, such as the specific degree of compression used in the socks in AE device therapy, compliance with AE device therapy, and follow-up of patients after discharge. Fifth, although we have made our best efforts to control for bias using multivariate models, moreover, subgroup analysis is limited by confounding factors, there are likely to be many other undiscovered factors. Finally, the study was subject to the standard limitations of large public databases, and so further studies—especially with a multicenter, large-scale, prospective design—are needed to remove these.

## Conclusions

Our findings demonstrated that the 28-day mortality and ICU mortality risks in the AE device therapy group were obviously lower than those in the no-AE device therapy group. This suggests that AE device therapy can improve the poor prognoses of patients with AMI complicated with type II DM, and that simple AE devices appear to be a fruitful direction for future research to improve poor prognoses. However, the present findings need to be confirmed in large prospective multicenter studies.

## Data availability statement

Publicly available datasets were analyzed in this study. This data can be found here: This data can be found here: The data were available on the MIMIC-IV website at https://mimic-iv.mit.edu/.

## Ethics statement

All procedures performed in studies involving human participants were in accordance with the Ethical Standards of the Institutional and National Research Committee and with the 1964 Helsinki declaration and its later amendments or comparable Ethical Standards.

## Author contributions

XH and LZ created the study protocol, performed the statistical analyses, and wrote the first manuscript draft. MX conceived the study and critically revised the manuscript. SY and YY assisted with the study design and performed data collection. TH assisted with data collection and manuscript editing. HY assisted with manuscript revision and data confirmation. JL contributed to data interpretation and manuscript revision. All authors read and approved the final manuscript.

## Funding

This study was supported by Guangdong Provincial Key Laboratory of Traditional Chinese Medicine Informatization (2021B1212040007).

## Conflict of interest

The authors declare that the research was conducted in the absence of any commercial or financial relationships that could be construed as a potential conflict of interest.

## Publisher's note

All claims expressed in this article are solely those of the authors and do not necessarily represent those of their affiliated organizations, or those of the publisher, the editors and the reviewers. Any product that may be evaluated in this article, or claim that may be made by its manufacturer, is not guaranteed or endorsed by the publisher.
