# Corrigendum: Anti-embolism devices therapy to improve the ICU mortality rate of patients with acute myocardial infarction and type II diabetes mellitus

**DOI:** 10.3389/fcvm.2023.1143652

**Published:** 2023-03-09

**Authors:** Xiaxuan Huang, Luming Zhang, Mengyuan Xu, Shiqi Yuan, Yan Ye, Tao Huang, Haiyan Yin, Jun Lyu

**Affiliations:** ^1^ Department of Neurology, The First Affiliated Hospital of Jinan University, Guangzhou, China; ^2^ Department of Clinical Research, The First Affiliated Hospital of Jinan University, Guangzhou, China; ^3^ Department of Intensive Care Unit, The First Affiliated Hospital of Jinan University, Guangzhou, China; ^4^ Guangdong Provincial Key Laboratory of Traditional Chinese Medicine Informatization, Guangzhou, China

**Keywords:** anti-embolic therapy, acute myocardial infarction, type II diabetes mellitus, mortality, ICU

A Corrigendum on Anti-embolism devices therapy to improve the ICU mortality rate of patients with acute myocardial infarction and type II diabetes mellitus By Huang X, Zhang L, Xu M, Yuan S, Ye Y, Huang T, Yin H, Lyu J. (2022) Front. Cardiovasc. Med. 9:948924. doi: 10.3389/fcvm.2022.948924.

**Error in Figure/Table**

In the published article, there was an error in Table 2 “Analysis of the associations between AE-device therapy and outcomes.” as published. We did not update the latest table in the final correction process, which resulted in its inconsistency with the text in the two sections of the abstract and results. The latest data should be “Adjusted: ICU mortality: HR = 0.48, 95% CI = 0.24–0.96, *P* = 0.039; Day 28 mortality: HR = 0.50, 95% CI = 0.27–0.90, *P* = 0.021”, **not** “Adjusted: ICU mortality: HR = 0.46, 95% CI = 0.23–0.93, *P* = 0.030; Day 28 mortality: HR = 0.49, 95% CI = 0.27–0.89, *P* = 0.020” as in the uncorrected table. The corrected Table 2 appears below.

**Table 2:** Analysis of the associations between AE-device therapy and outcomes. Analysis of the associations between AE-device therapy and outcomes.

	Non-AE device therapy	AE-device therapy	*P*-value
	HR (95%CI)	HR (95%CI)	
ICU Mortality			
Unadjusted	Reference	0.32 (0.22,0.48)	<0.001
Adjusted	Reference	0.48 (0.24,0.96)	0.039
Day 28 Mortality			
Unadjusted	Reference	0.44 (0.31,0.62)	<0.001
Adjusted	Reference	0.50 (0.27,0.90)	0.021

Analysis of the associations between AE-device therapy and outcomes. HR, hazard ratio; CI, confidence interval.

Models were derived from Cox proportional hazards regression models.

Model I was not adjusted for covariates.

Model II covariates were adjusted for Age, Weight, Ethnicity, Gender, First_careunit, APSIII, Anion_Gap, Heart_rate_mean, CKMB, WBC, Respiratory_rate_mean, Mbp_mean, SpO2_mean, Temperature_mean, Troponin_T_Max, Hemoglobin, Glucose_max, INR, Platelet, Potassium, Creatinine, Urea_Nitrogen, ALT, Urine_output, Lactate, Anti_Embolic, Antiplatelet, Anticoagulation, Congestive_heart_failue, Renal_disease, Malignant_cancer, Liver_disease, PCI, CABG, Ventilator, Vasopressor, CRRT, Peripheral_vascular_disease, Cerebrovascular_disease, Chronic_pulmonary_disease, Hypertensionid

The authors apologize for this error and state that this does not change the scientific conclusions of the article in any way. The original article has been updated.

**Text Correction**

In the published article, there was an error. In the **Abstract**, the HR, 95% CI, and *P*-values corresponding to 28-day mortality are incorrectly written as those corresponding to ICU mortality and the HR, 95% CI, and *P*-values for ICU mortality are written as those corresponding to 28-day mortality and here should use HR instead of OR. A correction has been made to **Abstract-“Results”**, Line 3–6 of the first paragraph. This sentence previously stated:

“In the multivariate analysis, compared with no-AE device therapy, AE device therapy was a significant predictor of 28-day mortality (OR = 0.48, 95% CI = 0.24–0.96, *P* = 0.039) and ICU mortality (OR = 0.50, 95% CI = 0.27–0.90, *P* = 0.021).”

The corrected sentence appears below:

“In the multivariate analysis, compared with no-AE device therapy, AE device therapy was a significant predictor of ICU mortality (HR = 0.48, 95% CI = 0.24–0.96, *P* = 0.039) and 28-day mortality (HR = 0.50, 95% CI = 0.27–0.90, *P* = 0.021).”

The authors apologize for this error and state that this does not change the scientific conclusions of the article in any way. The original article has been updated.

**Text Correction**

In the published article, there was an error. In the **Results** sections of this paper, the HR, 95% CI, and *P*-values corresponding to 28-day mortality are incorrectly written as those corresponding to ICU mortality and the HR, 95% CI, and *P*-values for ICU mortality are written as those corresponding to 28-day mortality, and here should use HR instead of OR. A correction has been made to Section of **Results-“Cox Proportional-hazards Models”**, Line 3–6 of the first paragraph. This sentence previously stated:

“As listed in Table 2, compared with no-AE device therapy, AE device therapy was a significant predictor of 28-day mortality (OR = 0.48, 95% CI = 0.24–0.96, *P* = 0.039) and ICU mortality (OR = 0.50, 95% CI = 0.27–0.90, *P* = 0.021) after adjusting for covariates.”

The corrected sentence appears below:

“As listed in Table 2, compared with no-AE device therapy, AE device therapy was a significant predictor of ICU mortality (HR = 0.48, 95% CI = 0.24–0.96, P = 0.039) and 28-day mortality(HR = 0.50, 95% CI = 0.27–0.90, *P* = 0.021) after adjusting for covariates.”

The authors apologize for this error and state that this does not change the scientific conclusions of the article in any way. The original article has been updated.

